# *In vivo* dosimetry for total body irradiation: five‐year results and technique comparison

**DOI:** 10.1120/jacmp.v15i4.4939

**Published:** 2014-07-08

**Authors:** Reshma P. Patel, Alison J. Warry, David J. Eaton, Christopher H. Collis, Ivan Rosenberg

**Affiliations:** ^1^ Radiotherapy Physics Department University College London Hospitals NHS Foundation Trust London UK; ^2^ Radiotherapy Department Royal Free Hospitals NHS Foundation Trust London UK

**Keywords:** *in vivo* dosimetry, total body irradiation, radiotherapy

## Abstract

The aim of this work is to establish if the new CT‐based total body irradiation (TBI) planning techniques used at University College London Hospital (UCLH) and Royal Free Hospital (RFH) are comparable to the previous technique at the Middlesex Hospital (MXH) by analyzing predicted and measured diode results. TBI aims to deliver a homogeneous dose to the entire body, typically using extended SSD fields with beam modulation to limit doses to organs at risk. *In vivo* dosimetry is used to verify the accuracy of delivered doses. In 2005, when the Middlesex Hospital was decommissioned and merged with UCLH, both UCLH and the RFH introduced updated CT‐planned TBI techniques, based on the old MXH technique. More CT slices and *in vivo* measurement points were used by both; UCLH introduced a beam modulation technique using MLC segments, while RFH updated to a combination of lead compensators and bolus. Semiconductor diodes were used to measure entrance and exit doses in several anatomical locations along the entire body. Diode results from both centers for over five years of treatments were analyzed and compared to the previous MXH technique for accuracy and precision of delivered doses. The most stable location was the field center with standard deviations of 4.1% (MXH), 3.7% (UCLH), and 1.7% (RFH). The least stable position was the ankles. Mean variation with fraction number was within 1.5% for all three techniques. *In vivo* dosimetry can be used to verify complex modulated CT‐planned TBI, and demonstrate improvements and limitations in techniques. The results show that the new UCLH technique is no worse than the previous MXH one and comparable to the current RFH technique.

PACS numbers: 87.55.Qr, 87.56.N‐

## I. INTRODUCTION

Total body irradiation (TBI) is a well‐established technique used in conjunction with chemotherapy in the treatment of systemic malignant diseases. Radiation is given to prepare for bone marrow or stem cell transplant by reducing the number of viable cells and suppressing the recipient's immune system. Typically, large fields are used at extended source‐to‐surface distance (SSD) in a parallel opposed arrangement to irradiate the entire body, with the patient in a standing or lying position.^1^, ^2^, ^3^ Due to the variations in the body contour along a patient's length and variations in tissue density, especially in the lungs, the dose distributions resulting from treating with two large open parallel opposed lateral fields may be highly inhomogeneous. In order to deliver a homogeneous dose across the body and to limit doses to organs at risk, the beams are modulated using bolus, compensators or shielding. *In vivo* dosimetry with thermoluminescent dosimeters (TLDs) or semiconductor diodes allows entry and exit doses to be recorded during treatment and used to calculate delivered midline doses.^1^, ^2^, ^3^, ^4^, ^5^, ^6^ These verify the delivery of the prescribed dose and indirectly check the patient position. Diodes have the advantage of real‐time intrafraction feedback and possibility for immediate correction.^(7)^ Calibration of diodes should be performed in TBI treatment conditions, due to the dependence of response on beam spectrum and dose rate.^(8)^

More recently, treatment planning using computed tomography (CT) scans of the patient have been introduced, using either in‐house^(9)^ or commercial software.^10^, ^11^, ^12^, ^13^, ^14^ CT data allow accurate estimation of organ‐at‐risk doses such as the lung, which usually limits the maximum tolerable dose. Placement of bolus and compensation can also be more accurately predicted before the first fraction of a multifraction treatment, reducing the need for test doses or modification later in the course.^(6)^ However, *in vivo* dosimetry is still essential for the verification of delivered dose accuracy and consistency in comparison to planned values. The accuracy of *in vivo* measurements should be±5%,^(4)^ in order to assess the overall aim of total delivered dose being within±10% of that prescribed.

CT‐planned TBI was introduced clinically at the Middlesex Hospital, London (MXH) in 1989,^10^, ^11^ with the patient lying supine with their knees bent. Briefly, depth doses and profiles measured under TBI conditions were configured into a commercial planning system, including modifications to allow for large field sizes and SSDs. Compensators were optimized to deliver uniform dose for bilateral fields within nine predetermined CT slices (including shoulders, lungs, and umbilicus). The compensators were then manually interpolated along the whole body by use of wax bolus. In addition, PMMA slabs were used for the head and bolus for the legs, to build up to the effective separation. The plan was normalized by assigning 100% to the highest lung dose. Diodes were used to verify the delivered dose, at five points along the body.^10^, ^11^

**Figure 1:**
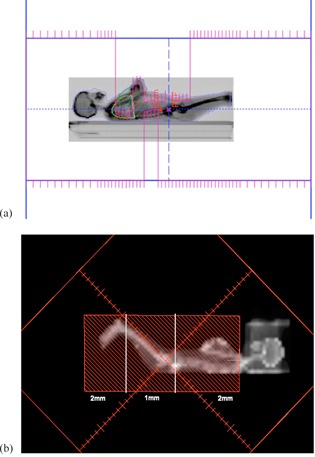
Beam's eye view (BEV) showing (a) UCLH MLC shielding and (b) RFH lead compensator sections.

**Figure 2:**
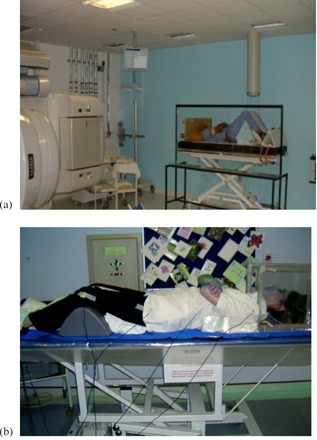
The UCLH treatment delivery setup (a) showing the spoiler screen and diode assembly; the RFH treatment setup (b) showing bolus around neck, hands, shoulders, and legs and the diode positions.

In 2005, when the Middlesex Hospital was decommissioned and merged with UCLH, both UCLH and the RFH introduced updated CT‐planned TBI techniques, based on the old MXH technique. More CT slices and *in vivo* measurement points were used by both; UCLH introduced a beam modulation technique using MLC segments, while RFH updated to a combination of lead compensators and bolus (Figs. 1 and 2).

The aim of this study was to review the *in vivo* diode measurements from both centers over five years of treatments, and compare them to the previous MXH technique in terms of the accuracy and consistency of delivered dose. Accuracy, reproducibility between fractions, and variations at different anatomical locations were assessed in terms of percentage dose differences between calculated and measured doses and their standard deviations.

## II. MATERIALS AND METHODS

### A. Treatment planning

#### A.1 UCLH

In the ‘new’ UCLH technique, a full CT dataset is acquired, extending from head to thighs, with a slice thickness of 10 mm and no additional bolus, and entered into the standard treatment planning system (Oncentra Master Plan, OMP, Nucletron, Veenendaal, The Netherlands). A TBI‐specific 10 MV machine was commissioned in OMP, using a pencil beam algorithm, to reproduce the measured dose distribution at the TBI treatment distance, including the effect of the beam spoiler (see below) on the buildup region. The treatment planning consists of adding MLC segments to shield or boost specific regions in the thorax, abdomen, and pelvis in order to improve the dose homogeneity, ensure the marrow producing bones are covered by 90%−110% of the prescription dose level, and ensure dose limits to lungs and kidneys are met (e.g., 50% and 10% of respective volumes to receive less than 100% of prescription dose) (Fig. 1(a)). The head and neck are compensated with PMMA slabs and bolus, and legs with bolus alone, as in the MXH technique. The plan is normalized to give 100% of the prescription dose to the midpoint at the level of the umbilicus. The accuracy of the TPS calculations was validated through planning a whole body anthropomorphic phantom (RANDO, The Phantom Laboratory, Salem, NY) and comparing predicted doses to TLD readings within the phantom.

Expected diode doses are calculated from the final treatment plan at a depth of 1.5 cm from the patient's external contour (corresponding to the inherent buildup of the detectors) at several relevant anatomical positions.

#### A.2 RFH

In the RFH technique, a CT dataset is used extending from head to knees with a slice thickness of 10−20 mm. A PMMA headbox, and bolus around the neck, hands, and between the legs are included in the scan. These data are used in Pinnacle treatment planning system (Philips, Amsterdam, The Netherlands) to produce a homogeneous treatment plan by the addition of lead compensators and further bolus, as required. The standard clinical 10 MV treatment beam model (using collapsed cone convolution) is used for calculations; the spoiler screens are simulated using dummy contours within the images to mimic the treatment setup. Typically, several different thicknesses of rectangular lead compensators are used to modulate the incident radiation intensity along the cranial–caudal axis (CCA) of the patient, with the compensator edges aligned in the axial direction (Fig. 1(b)). Extra PMMA may be added to the headbox, and extra bolus to the kidneys, under the arms (to cover the lungs), and to extend the headbox over the shoulders. A homogeneity of±5% is aimed for at midline, but±10% is acceptable, as long as dose volume constraints to the lungs and kidneys are achieved (e.g., 50% and 10% of respective volumes to receive less than 100% of prescription dose). Expected diode doses are calculated from the final plan at a depth of 2 cm from the patient's external contour, corresponding to the diodes' inherent buildup.

### B. Treatment delivery

#### B.1 UCLH

The patient is set up supine on the free‐standing TBI couch and the CCA length is measured in order to obtain the correct knee inflection to ensure feet are well within the field. The patient couch is rotated by 180° half way through the treatment. For each side, a sagittal laser 3.55 m from isocenter (4.55 m from the source) is used to align the patient midline perpendicular to the beam axis. Patient positioning is completed using the lateral tattoos at field center and with measurement of external anatomical landmarks. In addition, a zero dose MLC setup light field is used to check the arm positions and the couch height. A 15 mm thick PMMA beam spoiler screen is then placed in front of the patient at a distance of 0.25 m from the patient skin (Fig. 2(a)). The patient is instructed to keep as still as possible during the treatment and monitored using CCTV cameras from the control area. Treatment fields are delivered at a midline dose rate of about 30 cGy/min at 5 cm deep.

#### B.2 RFH

The patient is set up lying supine with bent knees on the dedicated TBI couch with their midline aligned to a laser 3.17 m from the isocenter (4.17 m from the source) (Fig. 2(b)). The large 25 mm thick PMMA spoiler screen is placed abutting the couch. The lead compensators are fixed to the block tray of the linac, using the light field shadow to align the edges. The treatment fields are delivered in four equal parts per side, owing to machine limitations on the maximum monitor units per beam. Diode readings are quickly analyzed after each of the four subfields on each side, and the couch is rotated by 180° half way through the treatment. The midline dose rate is 20−25 cGy/min.

### C. *In vivo* dosimetry

#### C.1 UCLH

The *in vivo* dosimetry system at UCLH consists of a 12 channel electrometer (which displays real‐time accumulated dose for each delivered treatment fraction and an interface which allows users to write comments about the treatment) connected to 12 semiconductor diodes with an inherent water‐equivalent buildup thickness of 1.5 cm (EDP‐15, InViDos Scanditronix Wellhofer AB, version 1.2, IBA Wellhofer, Belgium). The system is calibrated every six months under TBI conditions, and a predefined standard temperature correction is automatically applied by the InViDos software to the diode measurements when used on the patient. The data are then stored on a shared network drive and can be accessed at a later point for retrospective review. During treatment, diodes are placed at anatomical positions such as head, shoulder, thorax, center, knees, and ankles for both the left and right sides of the patient. The locations are chosen at positions easy to set up during treatment, away from MLC and field edges, and in an area of the field that does not have high dose gradients (based on the plan). Measured values are a sum of dose contributions from the entrance field and exit field doses for each fraction.

#### C.2 RFH

The RFH *in vivo* dosimetry system consists of up to 20 semiconductor diodes connected via infrared link to a base station (rf‐IVD2, Sun Nuclear, Melbourne, FL). *In vivo* dosimetry is used on every fraction to provide feedback on the delivered doses. Diodes are placed by measuring distances along the patient from the beam central axis. Each diode contains a temperature sensor to automatically compensate for variations in temperature when placed on the patient's skin. These corrections are checked regularly as part of the periodic quality assurance program. Calibration of the system is performed in TBI conditions (at extended SSD, behind the screen and with 2 mm lead on the treatment head), but constancy is checked against a reference reading collected using these setup conditions: all diodes strapped to a 1 cm thick sheet of PMMA, 100 cm SSD, 100 MU, 40 cm×40 cm, before each fraction. Any diode with constancy readings differing from the reference value, corrected for daily output, by more than 2% are typically excluded from use for that treatment session. Diode readings are taken on each side of the patient, rotating each fraction through a subset of ten sites from head to ankles. Flat (QED, Sun Nuclear, 1115000, $1.9g/{\text{cm}}^{2}$ buildup) diodes are used for all sites except hands, where cylindrical (ISORAD, Sun Nuclear, 1163000, $1.6g/{\text{cm}}^{2}$ buildup) diodes are used owing to oblique entry surfaces. Entrance and exit readings for each subbeam are entered into an Excel spreadsheet (Microsoft, Redmond, WA) to give immediate feedback on doses delivered compared to doses expected at 2 cm depth from the planning system, to within 7%. Exit diode readings are corrected for overresponse, and expected readings for underestimation by the planning system, compared to phantom measurements with an ionization chamber. On the first fraction, TLDs are also used at all potential measurement points, with buildup provided by plastic container discs. Gross errors are investigated immediately, and systematic differences are reviewed after the third fraction, at which point the shielding can be modified, if needed, for the remainder of treatment. For example, additional thickness of lead may be added to the region covering the knees and ankles if readings are high in this area.

### D. Data analysis

The data accumulated over the years have led to a sizable dataset for analysis to compare techniques. The UCLH measured doses are stored in the InViDos database (as were the MXH measurements). The database is periodically saved and exported to a Microsoft ACCESS database. The diode data were filtered to remove erroneous readings (typically greater than 20% difference to the expected value), which after further investigation of the comments written by users in the InViDos system were found to be due to malfunction or mispositioning, rather than a true difference in treatment delivery. The datasets amalgamated pediatric and adult patients of all sizes, ages, and gender. Single fraction patients were excluded because they would not be useful when analyzing time trends over several treatment fractions. RFH data were extracted from individual spreadsheets using a custom macro, averaged across both beam directions, and filtered to remove single fraction treatments.

## III. RESULTS

The total number of patients included in the analysis for the MXH and UCLH techniques were 162 and 135, respectively. The filtering mentioned in the previous section resulted in 1162 (444) data points being removed from a total of 19828 (9793) for the MXH (and UCLH) technique, respectively. This corresponds to about 6% of the points and should not influence the analysis. The number of patients included in the RFH analysis was 66, corresponding to 4106 data points. Erroneous readings were predominantly removed at the time of treatment review.

### A. Accuracy

**Figure 3:**
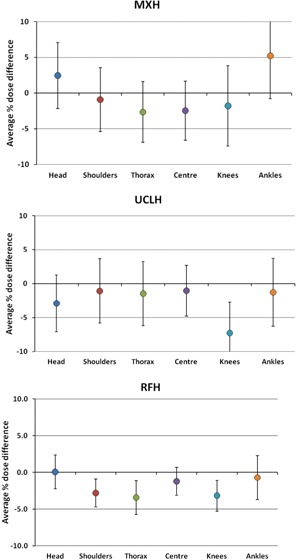
Percentage differences between measured and expected diode doses, averaged across all patients for each anatomical site, for the three techniques discussed in the text. Error bars represent 1 SD.

Figure 3 shows the percent variation between measured and expected diode doses, averaged across the whole population and over all fractions, for all monitored anatomical sites, for the MXH, UCLH, and RFH techniques. Variation within the population is indicated by the standard deviation (SD) of these values. The most stable position was the center, with SD of 4.1%, 3.7%, and 1.7%, respectively, for the three techniques. The least stable position was the ankles, with SD of 6.0%, 5.0%, and 2.9%, respectively.

### B. Precision

**Figure 4:**
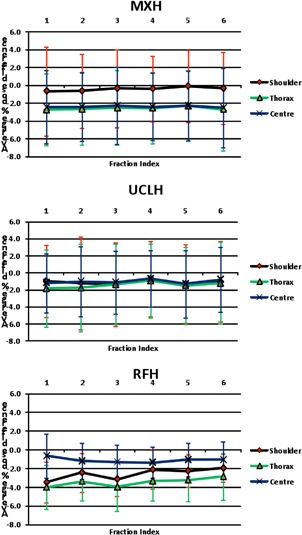
Percentage differences between expected and measured diode doses, averaged across all patients for each treatment fraction, for three anatomical sites for the three techniques discussed in the text. Error bars represent 1 SD.

Figure 4 shows the percent variation between expected and measured diode doses, averaged across the whole population as a function of treatment fraction, at the three most significant anatomical sites (shoulders, thorax, and center). The interfractional variation to these sites was 0.6%, 0.9%, and 1.5% for the three techniques: MXH, UCLH, and RFH, respectively.

## IV. DISCUSSION

Accuracy is a measure of how close the delivered dose is to the expected dose. For all three techniques, the *in vivo* measured doses were within±10% of the expected doses, which is clinically acceptable. Compared to the MXH technique, the UCLH technique gave similar values for the planned sites of shoulder, thorax, and center, with average measured doses approximately 1% closer to expected values. Head, knees, and ankles, however, which are still compensated using PMMA and bolus following the MXH technique, showed a decrease of 5%−6% relative to MXH differences. The probable reason for this consistent discrepancy is the different way the plans were normalized. The MXH technique forced 100% of the prescription dose at the maximum of the compensated lung slice. The effective separation to be applied to the head and legs was derived from this normalization. The UCLH technique is normalized to midline at the level of the umbilicus and the actual separation there was applied to the head and legs. These different approaches must have produced a difference of about 2.5 cm in required separation.

RFH values showed similar agreement, within 4%, for all sites including extremities. The variation across the patient population was also lower than the UCLH techniques. This may be a result of scanning down below the knees and including bolus within the computerized treatment plan. Shoulder and thorax (lung) values are systematically lower than those at the center (waist). Shoulder and hip regions are often covered by little or no lead compensation, since they are typically the widest separation. Therefore, the irradiation conditions are most different to how the diodes are calibrated, under 2 mm of lead, as typically used over the center. As expected, the most stable position was the center, while the ankles were the least stable, owing to mobility and difficulties in positioning accurately. The measured diode dose data for all techniques are systematically lower than the doses derived from the TPS, but these are within uncertainties in calculation and measurement, so may not be significant.

Precision is how consistently the measured dose is reproduced, analyzed by considering variations as a function of the treatment fractions. Changes over the course of treatment reflects the complexity of identifying suitable positions for the detectors (regions of low dose gradient) and the challenges of consistent patient and diode positioning between fractions. All three techniques showed similar low variation during the treatment, with no discernible trends with fraction number for the MXH and UCLH techniques. For the RFH approach, some increase is seen for the shoulder and thorax doses, but this is closer to the expected values and probably reflects increased experience with patient setup as the treatment progresses.

Comparison to other studies is dependent on the differences in equipment and techniques used. However, a number of other authors have published cohort analyses using CT planning and diode *in vivo* dosimetry. Mangili et al.^(6)^ treated 229 patients over five years, including 115 using the CadPlan commercial TPS. Agreement of diode measurements at waist and ankles was 0.0%±4.5%, reducing to ±3.5% with CT planning. Bloemen‐van Gurp et al.^(15)^ measured five points from neck to ankle using MOSFETs for ten CT‐planned patients. Modulation within the plans was achieved using MLC segments, and mean agreement with planned midline doses were −0.5%. Ramm et al.^(16)^ used a couch translation technique with CT planning and diode measurements at four points from head to waist, with 80% of readings within 5% of expected values. Lancaster et al.^(17)^ treated 86 patients over seven years with a CT‐planned technique combining AP/PA and lateral fields. Diodes placed at five points from head to pelvis for the first lateral beam fraction to measure mean exit dose were −4.3%±7.8% compared to planned values. Values reported in the current study are at least comparable and, in some cases, better compared to these other authors. Both modern techniques at UCLH and RFH are more closely related to the MXH technique than any other published method; therefore, the comparisons made above between the three are the most useful.

Plans calculated in the Pinnacle TPS using a standard beam model have shown good agreement with doses measured in phantom, including modeling of lung shielding by arms, complex aluminum compensators or MLC blocking of head and lung.^12^, ^13^ Lavallée et al.^(14)^ found improved accuracy with a specific TBI beam model, but this was mainly in the area of out‐of‐field dose, which was significant for the AP/PA field translating couch technique being used. In addition, the TBI model was unable to account for the first centimeter of the depth dose curve with the beam spoiler in place, owing to the standard model‐based nature of the superposition‐convolution algorithms. Out‐of‐field doses are not significant for the UCLH/RFH techniques of lateral fields at extended SSD, therefore no additional modification of the beam model is warranted.

## V. CONCLUSIONS

The extensive and consistent *in vivo* measurements of TBI techniques increase the confidence in the dosimetry and delivery of this complex technique. It also gave good evidence that new techniques were equal or superior to old long‐established ones.

## ACKNOWLEDGMENTS

The authors would like to acknowledge Mr. Derek D’Souza, the Head of Radiotherapy Physics, UCLH, for providing support and leadership. Acknowledgements to Christopher Stacey, who was instrumental in the development and application of the MLC‐based treatment technique at UCLH and, as a result, made this comparison work possible. Personal thanks to Andrew Roberts who played a significant role in providing database advice, support, and basic training.

## References

[1] Van Dyk J , Galvin JM , Glasgow GP , Podgorsak EB . AAPM Report 17: The physical aspects of total and half body irradiation. New York: American Institute of Physics; 1986.

[2] Gerrard GE , Vail A , Taylor RE , et al. Toxicity and dosimetry of fractionated total body irradiation prior to allogeneic bone marrow transplantation using a straightforward radiotherapy technique. Clin Oncol (R Coll Radiol). 1998;10(6):379–83. 9890540 10.1016/s0936-6555(98)80034-4

[3] Harden SV , Routsis DS , Geater AR , et al. Total body irradiation using a modified standing technique: a single institution 7 year experience. Br J Radiol. 2001;74(887):1041–47. 11709470 10.1259/bjr.74.887.741041

[4] Greig JR , Miller RW , Okunieff P . An approach to dose measurement for total body irradiation. Int J Radiat Oncol Biol Phys. 1996:36(2):463–68. 8892472 10.1016/s0360-3016(96)00268-4

[5] Ribas M , Jornet N , Eudaldo T , et al. Midplane dose determination during total body irradiation using in vivo dosimetry. Radiother Oncol. 1998;49(1):91–98. 9886703 10.1016/s0167-8140(98)00094-2

[6] Mangili P , Fiorino C , Rosso A , et al. In‐vivo dosimetry by diode semiconductors in combination with portal films during TBI: reporting a 5‐year clinical experience. Radiother Oncol. 1999;52(3):269–76. 10580875 10.1016/s0167-8140(99)00104-8

[7] Sánchez‐Doblado F , Terrón JA , Sánchez‐Nieto B , et al. Verification of an on line semiconductor dosimetry system for TBI with two TLD procedures. Radiother Oncol. 1995;34(1):73–77. 7792402 10.1016/0167-8140(94)01490-t

[8] Jornet N , Ribas M , Eudaldo T . Calibration of semiconductor detectors for dose assessment in total body irradiation. Radiother Oncol. 1996;38(3):247–51. 8693106 10.1016/0167-8140(95)01677-5

[9] Sánchez‐Nieto B , Sánchez‐Doblato F , Terrón JA . A CT‐aided PC‐based physical treatment planning of TBI: a method for dose calculation. Radiother Oncol. 1997:42(1):77–85. 9132830 10.1016/s0167-8140(96)01857-9

[10] Planskoy B , Bedford AM , Davies FM , Tapper PD , Loverock LT . Physical aspects of total‐body irradiation at the Middlesex Hospital (UCL group of hospitals), London 1988‐1993: I. Phantom measurements and planning methods. Phys Med Biol. 1996:41(11):2307–26. 8938028 10.1088/0031-9155/41/11/005

[11] Planskoy B , Tapper PD , Bedford AM , Davies FM . Physical aspects of total‐body irradiation at the Middlesex Hospital (UCL group of hospitals), London 1988‐1993: II. In vivo planning and dosimetry. Phys Med Biol. 1996:41(11):2327–43. 8938029 10.1088/0031-9155/41/11/006

[12] Abraham D , Colussi V , Shina D , Kinsella T , Sibata C . TBI treatment planning using the ADAC Pinnacle treatment planning system. Med Dosim. 2000:25(4):219–24. 11150693 10.1016/s0958-3947(00)00049-2

[13] Hui SK , Das RK , Thomadsen B , Henderson D . CT‐based analysis of dose homogeneity in total body irradiation using lateral beam. J Appl Clin Med Phys. 2004:5(4):71–79. 10.1120/jacmp.v5i4.1980 PMC5723515 15738922

[14] Lavallée M‐C , Gingras L , Chrétien M , Aubin S , Côté C , Beaulieu L . Commissioning and evaluation of an extended SSD photon model for PINNACLE3: an application to total body irradiation. Med Phys. 2009:36(8):3844–55. 19746817 10.1118/1.3171688

[15] Bloemen‐van Gurp EJ , Mijnheer BJ , Verschueren TAM , Lambin P . Total body irradiation, toward optimal individual delivery: dose evaluation with metal oxide field effect transistors, thermoluminescence detectors, and a treatment planning system. Int J Radiat Oncol Biol Phys. 2007:69(4):1297–304. 17881143 10.1016/j.ijrobp.2007.07.2334

[16] Ramm U , Licher J , Moog J , et al. In vivo dosimetry with semiconducting diodes for dose verification in total‐body irradiation. A 10‐year experience. Strahlenther Onkol. 2008:184(7):376–80. 19016037 10.1007/s00066-008-1823-5

[17] Lancaster CM , Crosbie JC , Davis SR . In‐vivo dosimetry from total body irradiation patients (2000 ‐ 2006): results and analysis. Australas Phys Eng Sci Med. 2008:31(3):191–95. 18946976 10.1007/BF03179343

